# Comparison of the Indentation Processes Using the Single Indenter and Indenter Array: A Molecular Dynamics Study

**DOI:** 10.1186/s11671-022-03686-4

**Published:** 2022-05-02

**Authors:** Yanquan Geng, Jiqiang Wang, Zihan Li, Yongda Yan, Jingran Zhang, Yang Gan

**Affiliations:** 1 Key Laboratory of Micro-Systems and Micro-Structures Manufacturing of Ministry of Education, Harbin Institute of Technology, Harbin, 150001 Heilongjiang People’s Republic of China; 2 Center for Precision Engineering, Harbin Institute of Technology, Harbin, 150001 Heilongjiang People’s Republic of China; 3 College of Mechanical and Electric Engineering, Changchun University of Science and Technology, Changchun, 130000 Jilin People’s Republic of China; 4 School of Chemistry and Chemical Engineering, Harbin Institute of Technology, Harbin, 150001 People’s Republic of China

**Keywords:** Nanoindentation, Periodic nanostructure, Single indenter, Indenter array, Indention morphology, Molecular dynamics

## Abstract

Fabrication of periodic nanostructures has drawn increasing interest owing to their applications of such functional structures in optics, biomedical and power generation devices. Nano-indentation technique has been proven as a method to fabricate periodic nanostructures. In this study, the molecular dynamic simulation approach is employed to investigate the nano-indentation process for fabricating periodic nano-pit arrays using a single indenter and an indenter array. The morphologies of indentations that machined using these two kinds of indenters are compared. The indentation force and the defect evolution during the nano-indentation process are further studied. Results show that indentation morphologies obtained by single indenter are mainly depended on the spacing of indenters, and a nano-pit array with a better shape and consistency can be obtained easier using the indenter array compared with using a single indenter. The stacking faults and dislocations induced by indentation are depended on the spacing of the indenters. Our findings are significant for understanding the differences of indentation processes using a single indenter and an indenter array and machining a high-quality periodic nano-pit array with high machining efficiency.

**Graphical Abstract:**

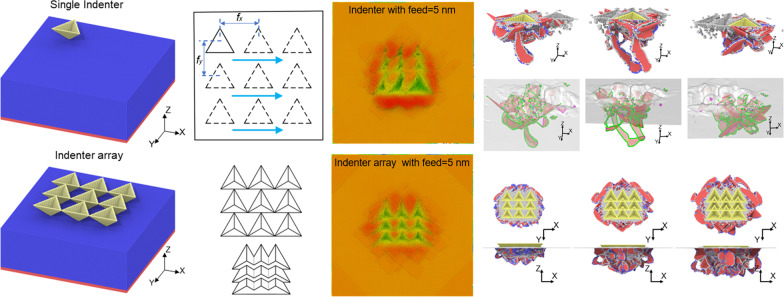

## Introduction

Periodic nanostructures have drawn significant attentions in the recent years owing to their applications in fields of Raman detection [1], hydrophobic structure [2], and biosensing [3]. Several micro and nanofabrication techniques, for instance, photolithography [4], electron-beam lithography (EBL) [5], nanoimprinting [6] and tip-based nanomachining [7], thus have been utilized to machine nanostructures. However, these methods are impeded by some disadvantages. The resolution of photolithography is limited by the wave length and the operation is complicated. The machining efficiency for EBL is relatively low, moreover, it is difficult to machine a large-scale nanostructure. For the nanoimprinting approach, the crucial issue is how to machine a template with a high-precision nanostructure.

Nanoindentation technique has been proven as an alternative than the aforementioned method to fabricate periodic nanostructures [8]. Chang et al. [9] machined nano-pit arrays on Au film using a Berkovich indenter, and the obtained structures were utilized as substrates for Raman detection. Yan et al. [10] proposed a method, which combines indentation and ultra-precision turning, to fabricate structural surfaces. Jeon et al. [11] developed an advanced indentation processing system and achieved the maximum processing frequency of 10 Hz. Moreover, in our previous study, a probe-based force-controlled nanoindentation system with an axisymmetric four-beam spring is developed to overcome the problem of the various lateral force generated for different scratching directions [8]. It can be found that the indentation array obtained in our previous study was conducted by a single indenter, and the fabricated structure in the edge area is inconsistent with even a relatively large feed value of 3 μm. Therefore, a more feasible indentation strategy is needed to fabricate periodic nanostructures with good consistency and high efficiency.

Structural tool, a tool has multi-tip with a given spacing, has be used to fabricate multi-nanogrooves with good consistency by only single-scratch process, which can also improve the efficiency of the machining process [12–16]. Thus, it can be inferred that a structural indenter with a tip array could be used to obtain nanostructures with good consistency for even a small period. However, no previous works focusing on the indentation process with an indenter array are found up to now. Furthermore, molecular dynamic (MD) simulation approach has been proved as a powerful tool to study the nanomachining process [17–20]. It can be used to simulate the morphology of the machined structure, the structural characteristics of the sample and the subsurface defects at the atomic scale [21, 22].

Therefore, in this study, the nano-indention processes with a single indenter and an indenter array were investigated by using MD simulation approach. The effect of the spacing of the indenter on the indentation morphology was investigated. Furthermore, the indentation force as well as the evolution of the subsurface defects caused by these two kinds of indenters were also studied.

## Methods

**Fig. 1:**
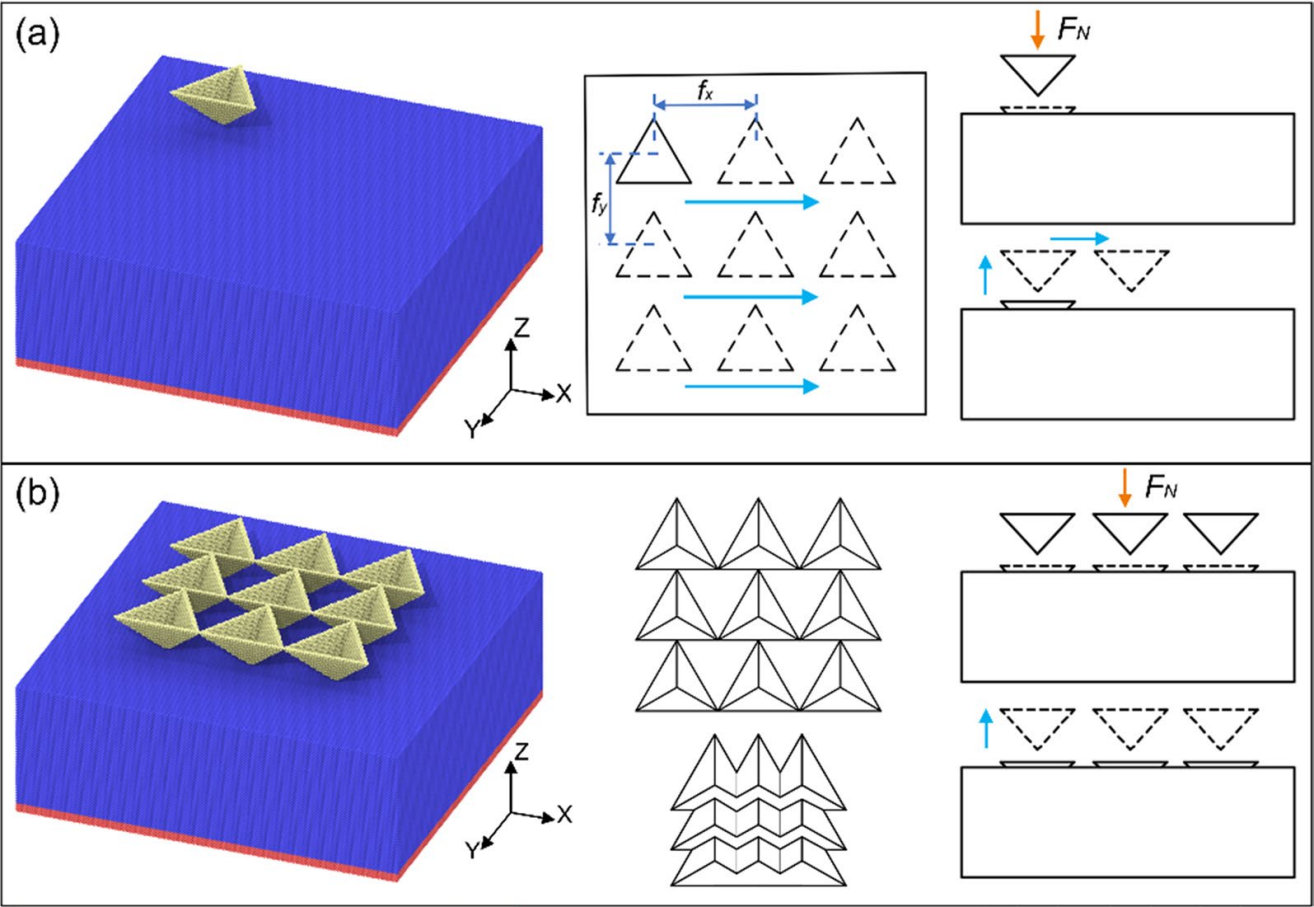
MD simulation models **a** single indenter, **b** indenter array

The MD model of the indentation process is shown in Fig. 1, which includes a single triangular pyramid indenter or a triangular pyramid indenter array and a single crystal copper specimen. The dimensions of the single crystal copper sample are 47 nm × 47 nm × 16 nm, and the three crystal orientations are *x* [1 0 0], *y* [0 0 1], *z* [0 0 1]. Thus, the crystal plane of the copper surface is (1 0 0) in this study. Periodic boundary conditions are used in X and Y directions, and the fixing layer is applied at the bottom of the workpiece. The diamond indenters are defined as rigid bodies. Cu-Cu is described by EAM potential function, and C-Cu by Morse potential function. As shown in Fig. 1a, the single triangular pyramid indenter is adopted to impress into the sample surface with a pre-set normal load, and lift with a constant speed. Then, the indenter is controlled to move to next position with a feed distance along *x* direction. After three indentations, the indenter is controlled to return to the original position and feed in the *y* direction with a given value. Nine indentations are carried out in the whole process. While, the triangular pyramid indenter array contains 9 triangular pyramid indenters with the same geometry, as shown in Fig. 1b. The space distance between adjacent two triangular pyramid indenters is also defined as the feed distance. Nine indentation structures can be produced by one cycle of penetration and lifting process. In this study, two feed distances of 5 nm and 10 nm are selected for the MD simulation.

Before the indentation process, the sample is equilibrated in the NVT ensemble at 30K for 50 ps. The rest parameters are listed in Table 1. All the MD simulations are conducted using open source Large-scale Atomic/Molecular Massively Parallel Simulator (LAMMPS) software, and the Intel E5 36-core computer is adopted for the simulation, and the computation time for each simulation is about 20 h. The simulation results are analyzed by OVITO software [23].

**Table 1:** Simulation parameters

Parameters	Values
Atoms in workpiece	3,075,800
Atoms in tool	Single indenter: 5523 Indenter array: 49,707
Normal load	Single indenter: 441.8 nN Indenter array: 2863.1 nN (Spacing = 5 nm) 3578.9 nN (Spacing = 10 nm)
Spacing distance	5 nm, 10 nm

## Results and Discussion

### Nano-Pit Array Morphology

**Fig. 2:**
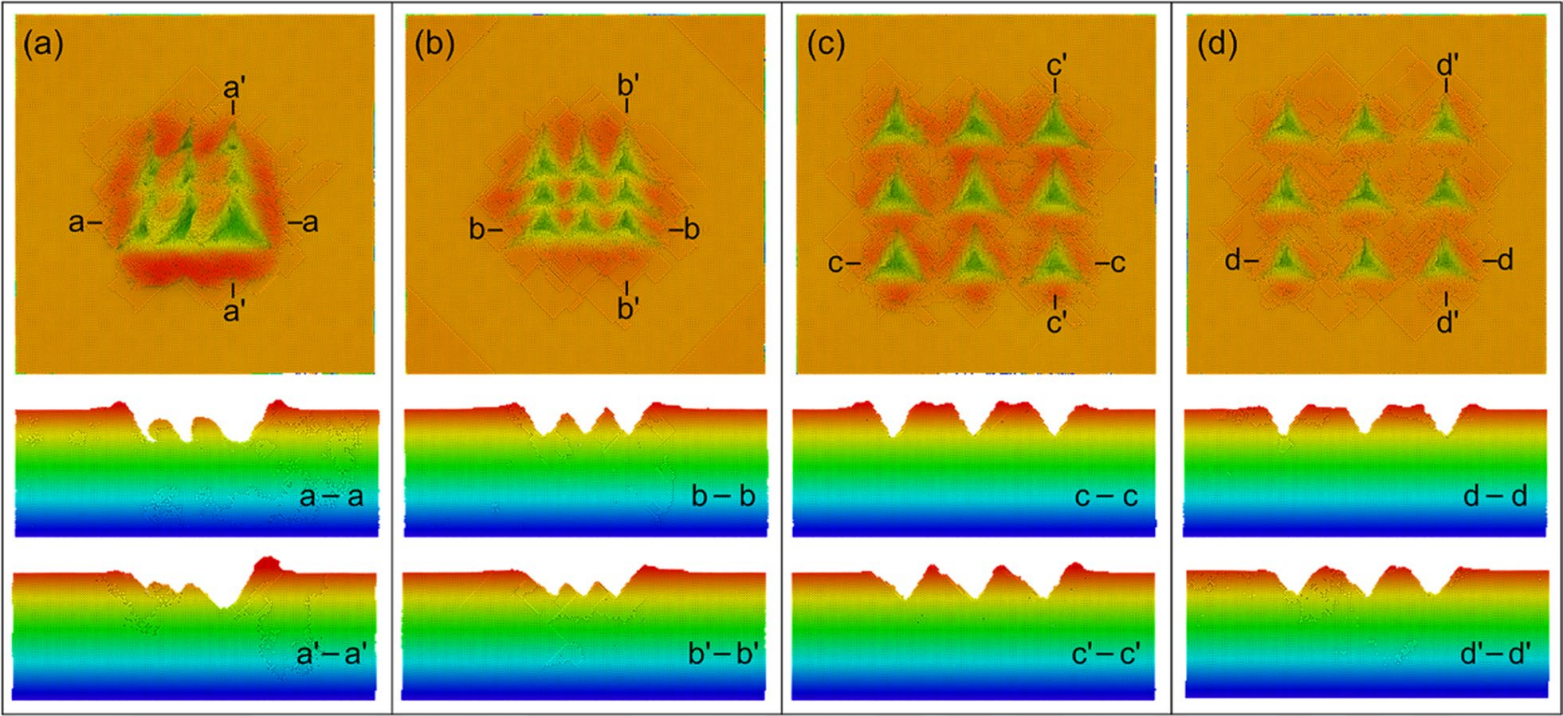
Indentation morphology **a** indenter with feed = 5 nm, **b** indenter array with feed = 5 nm, **c** indenter with feed = 10 nm, **d** indenter array with feed = 10 nm

The indentation morphologies obtained by the single indenter and indenter array with different feed distances are shown in Fig. 2. As shown in Fig. 2a, when the spacing is selected as 5 nm, a nano-pit array is formed by the single indenter. However, due to the influence of material accumulation, the morphology of the nano-pit varies greatly, and the size of the latter nano-pit is obviously larger than that of the previous one. In addition, a more complete pyramid pit structure can be formed for the last indentation. Thus, it can be concluded that is the nano-pit array with good consistency is difficult to be obtained by the point-by-point indentation process with a relatively small interval. While, it can be observed from Fig. 2b that nano-pit array with the feed distance of 5 nm can be fabricated, and the obtained nano-pit array shows good consistency. When the spacing distance is enlarged to 10 nm, due to the relatively large spacing value, the influence of the materials accumulation is small, and an array structure of triangular pyramidal nano-pits with consistent shape, depth and good periodicity can be machined by the single indenter, as shown in Fig. 2c. Huo et al. had investigated the nanoindentation process by using AFM tip [24]. They machined nanoindentation array with good periodicity, which shows an agreement with the present simulated results. For the case of indenter array, a nano-pit array with good consistency and periodicity can also be obtained with the spacing dictation of 10 nm, as shown in Fig. 2d. It can be found that the nano-pit array structure can be generated by the indenter array with only one pressing and lifting process, while, for the single indenter, it needs 9 times point by point indentation processes to fabricate the nano-pit array structure. It can be concluded that the use of indenter array can greatly improve the machining efficiency and machining quality.

It can also be found from the cross section in Fig. 2a that the material stacking is correspondingly serious when conducting point by point indentation process with the spacing distance of 5 nm, and the depth and width of the nanostructure increase successively with the processing times. In addition, it is obvious that the third extrusion has an effect on the first two extrusions, and the material bulges inside the nano-pit structure are inclined to one side. While, as shown in Fig. 2b, the material bulges inside the nano-pit structure obtained by indenter array with the spacing distance of 5 nm show good consistency and symmetry. When the spacing distance increases, as shown in Fig. 2c, d, the stacking effect weakens, and there is no significant difference between the indenter array and the nano-structure produced by point-by-point indentation, but the height of the material accumulation of the point-by-point indentation is higher, which is caused by the extrusion action of each indentation.

**Fig. 3:**
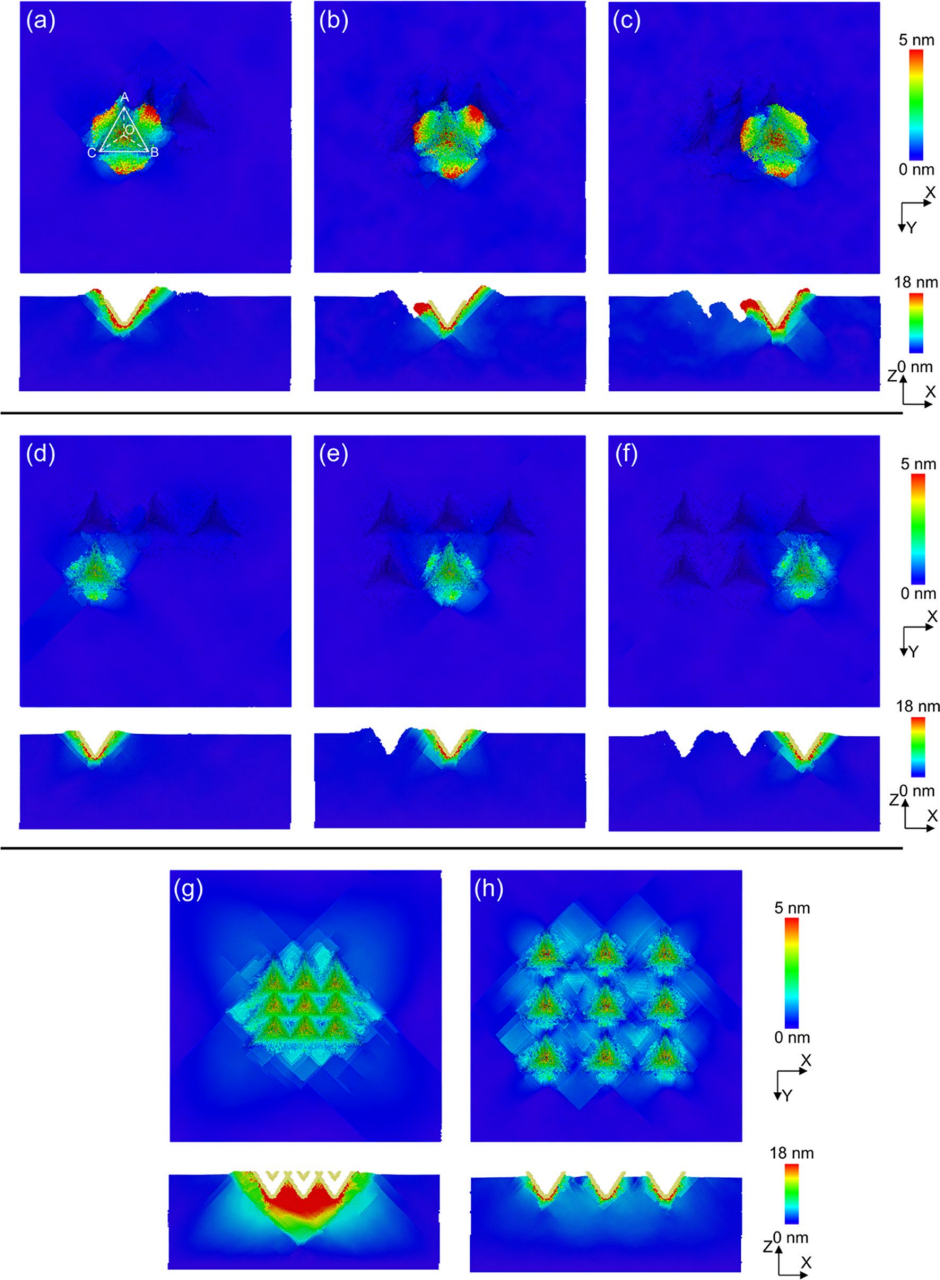
Atomic displacement diagram of nanoindentation **a** the fourth indentation of Single-indenter with feed = 5 nm, **b** the fifth indentation of Single-indenter with feed = 5 nm, **c** the sixth indentation of Single-indenter with feed = 5 nm, **d** the fourth indentation of Single-indenter with feed = 10 nm, **e** the fifth indentation of Single-indenter with feed = 10 nm, **f** the sixth indentation of Single-indenter with feed = 10 nm, **g** the multi-indenter indentation with feed = 5 nm, **f** the multi-indenter indentation with feed = 10 nm

In order to further study the morphology generation process, the changes of atomic displacements before and after pressing are also analyzed, which are distinguished by color gradient according to the height information, as shown in Fig. 3. Under the action of the indenter, the workpiece material is squeezed by each face of the indenter to flow in the direction of the normal vector of each face, and some materials are squeezed upward along the inclined face of the indenter to form the accumulation. When the spacing distance is selected as 5 nm, as shown in Fig. 3a, the workpiece material flows on both sides of the edge OA with the influences of surface OAB and surface OAC, resulting in serious deformation of the morphology of the previous indentation. In addition, the processed indentation morphology also has an effect on the material deformation of the latter indentation. The surface OAB of the indenter penetrates the second indentation, while, the surfaces OAC and OBC sides are unprocessed material, which results in the material on the side of the surface OAB easier to be deformed. Therefore, the atomic displacement on one side of the surface OAB is higher and mainly flows in the direction of the second indentation. It can also be found in Fig. 3b that the atoms near the surface OAC are concentrated toward the fourth indentation, while, the atoms near the surface OAB are concentrated toward the third indentation. As shown in Fig. 3c, the atomic displacement near surface OAB is relatively smaller for the sixth indentation. When the spacing distance is enlarged to 10 nm, the adjacent indentation influences each other less, and the workpiece material flows evenly to the three surfaces of the indenter. In addition, the atomic flow state for each indentation is consistent. For the case of the indenter array, it can be found for Fig. 3g that the indenter array can effectively reduce the overlapping effect of the adjacent indentations. The atomic flow trend of each structure is consistent. It can also be observed from the cross section that the atomic flow trend of the subsurface layer of the workpiece is symmetric along the center line and the atoms flows downward. In Fig. 3h, the material deformation at each indentation position is consistent, and each indenter has the same influence on the atomic flow.

### Indentation Force

**Fig. 4:**
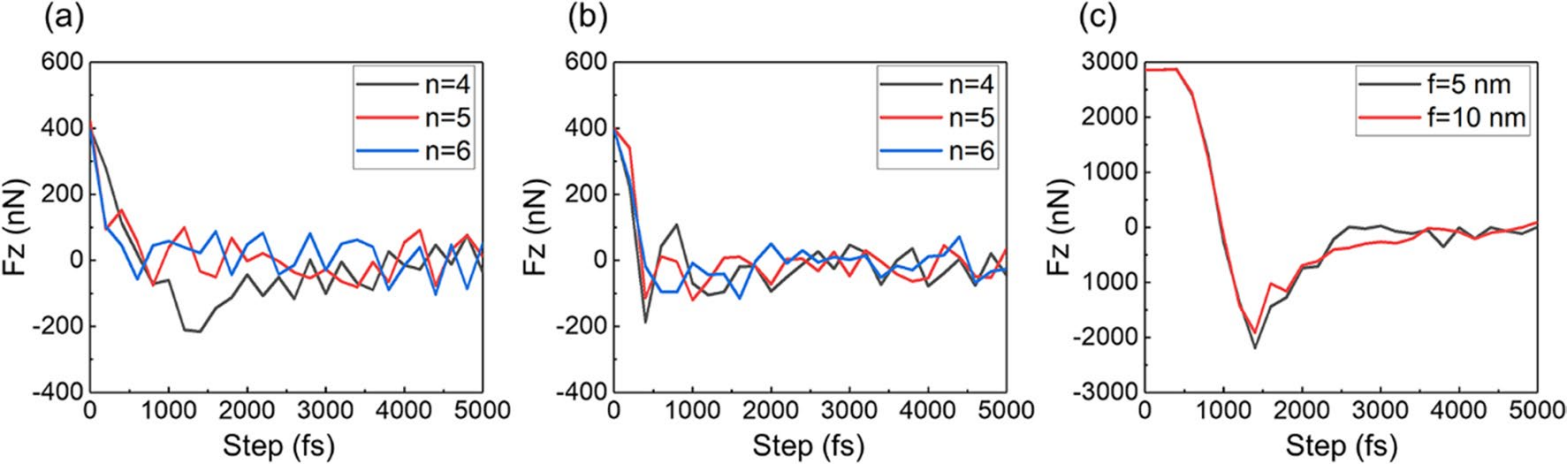
Indentation forces **a** single indenter indentation with feed = 5 nm, **b** Single indenter indentation with feed = 10 nm, **c** multi-indenter indentation

There are three directional forces in the nanoindentation process. While, due to the two indentation forces orthogonal to the direction of indenter movement, the force change is not obvious during the indentation process. Thus, the indentation force in Z-direction is extracted for discussion and analysis in this study. As shown in Fig. 4, the indentation force–time curves of fourth to sixth indentations is extracted for the single indenter, and the indentation force–time curves for one indentation are extracted for indenter array. In Fig. 4a, since the indenter is pressed under a constant load and pressed into the sample surface at a constant acceleration, the indentation force drops sharply when the indenter contacts the workpiece. When the indentation force decreases to zero, because the speed of the indenter is not zero, the indenter continues pressing down, currently the indentation force increases in the opposite direction. When the indentation force increases to the maximum, the velocity of the indenter decreases to zero. Due to recovery of the material, the indentation force decreases until the applied load is balanced with the sample reaction. Because of the applied normal load, the nanoindentation process is always accompanied by the nucleation and expansion of dislocations and the accumulation and release of atomic potential energy, and their fluctuations occur in the equilibrium process. For the fifth and sixth indentations, because the pressing area is located above the processed structure, the contact area between the material and the indenter is reduced and the workpiece material hardness are changed. Thus, the indentation forces of the fifth and sixth indentations along Z-direction are decreased compared with that of the fourth indentation. As shown in Fig. 4b, due to the relatively large spacing distance between the adjacent indentations, the structure overlapping effect is reduced, therefore, the change of indentation force is unobservable. Compared with the single indentation process, due to the relatively larger indentation force for the indenter array, the time to the equilibrium state is longer, as shown in Fig. 4c. When the indentation process reaches to a stable state, the fluctuation of indentation force of the indenter array is less than that of single indentation process, which may result from the dislocation interaction caused by each indenter impeding the continuous evolution during the indenter array pressing into the workpiece material.

### Defect Evolution During the Nano-Indentation Process

**Fig. 5:**
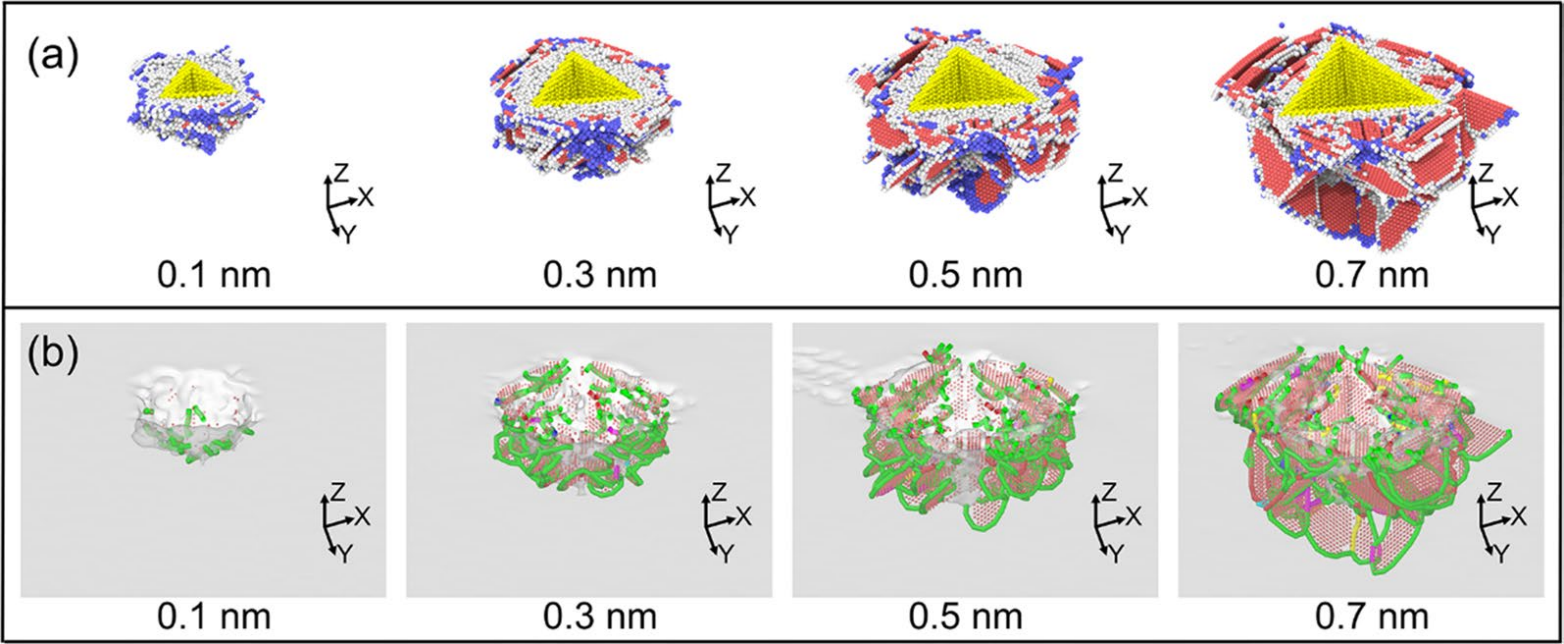
Dislocation structures generated in Single-Indenter indentation process **a** CNA images of workpiece, **b** DXA images of workpiece

In the nano-indentation process, the materials extrusion obtained by the indenter result in plastic deformation, which is realized by the nucleation and motion of dislocations [25]. Figure 5 shows the dislocation evolution in the first nano-indentation process of single-indenter, which is analyzed by CNA and DXA methods. The atoms are colored according to the calculated CNA values: the hexagonal close packed (HCP) atoms are red and the body centered cubic atoms are blue. Other type of atoms such as dislocation cores and surface atoms are white and the atoms in face-center cubic (FCC) structures are not displayed. Dislocation lines identified by DXA are colored according to their types: Shockley partial dislocation (green line), and stair-rod dislocation (purple line). Under the influence of the stress exerted by the indenter, the BCC atoms are formed at the depth of 0.1 nm in Fig. 5a. As the indenter continued to load, the slip system is activated in (1 1 1) and the dislocation atoms are nucleated and extended along the slip system. In addition, it can be seen from Fig. 5b that Shockley partial dislocation lines also existed, which takes the main part of them in the whole indentation process. When the indenter loaded to the deepest point, the stacking faults on these planes slide along burgers vector until intersection, forming a stair-rod dislocation lines to impede the movement of staking faults.

**Fig. 6:**
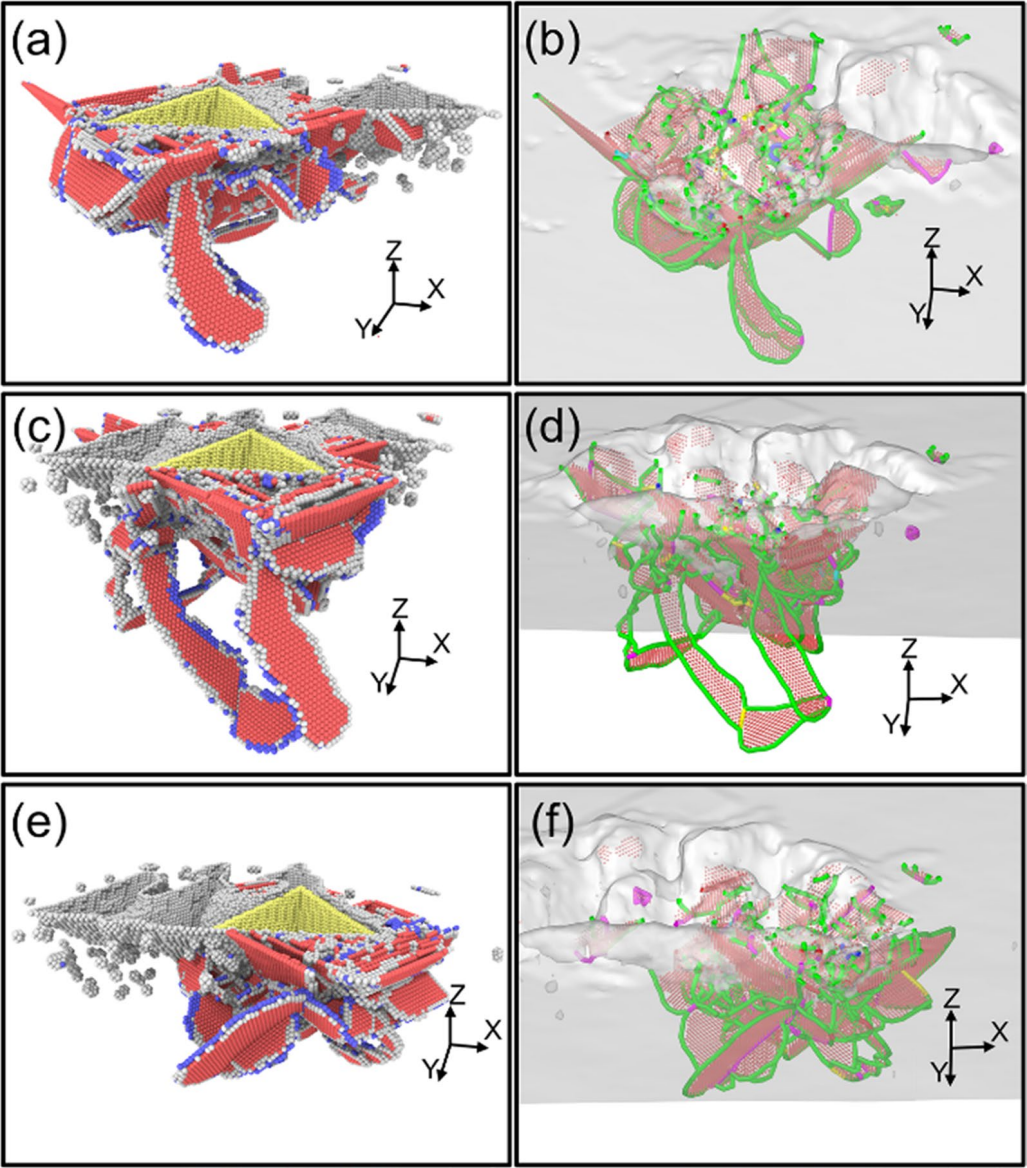
Dislocation structures generated in Single-Indenter indentation process with the spacing of 5 nm **a** CNA snapshot in the fourth indentation, **b** DXA snapshot in the fourth indentation, **c** CNA snapshot in the fifth indentation, **d** DXA snapshot in the fifth indentation, **e** CNA snapshot in the sixth indentation, **f** DXA snapshot in the sixth indentation

The dislocation distribution from the fourth to the sixth indentations of the single-indenter at the spacing of 5 nm is shown in Fig. 6. In Fig. 6a, unlike the first indentation, the dislocations generate under the extrusion between the OAB surface of the indenter and workpiece are hindered during the propagation process due to the pit structures formed from the first to the third indentation process, which results in a lower degree of defect evolution than that in the first indentation. Besides, the dislocation loop which is consisted of several Stacking fauls and screw dislocations are formed under the surface OBC. The screw dislocation is formed under the driving of the compression stress state of shear-slip zone [26]. As shown in Fig. 6b, stacking faults are wrapped by the Shockley dislocation lines and each stacking fault is connected by the Stair-rod dislocation line. The dislocation loop is dropping away from the bottom of the indenter. Moreover, the dislocation loop also could be observed beneath the surface OBC in the fifth indentation in Fig. 6c, d and the extent of the propagation is greater than that of the fourth indentation. This may be due to that when the indenter loading at the first time, a large number of stacking faults intersected, forming the hindrance to prevent further evolution in Fig. 5. However, under the influence of the machined pit, the propagation of the dislocations formed by the surfaces OAB and OAC are blocked so that the dislocations could be continue to propagate beneath the surface OBC, forming the dislocation loop. In Fig. 6e, f, dislocation distribution is also Fig. 1 quite different from the first time and the dislocation loop is also distributed on the side of the surface OBC. In addition, due to the unloading of the indenter, the deformation energy decreased the faults are gradually annihilated and the cluster atoms broke away from the stacking faults distributed in the subsurface layers.

**Fig. 7:**
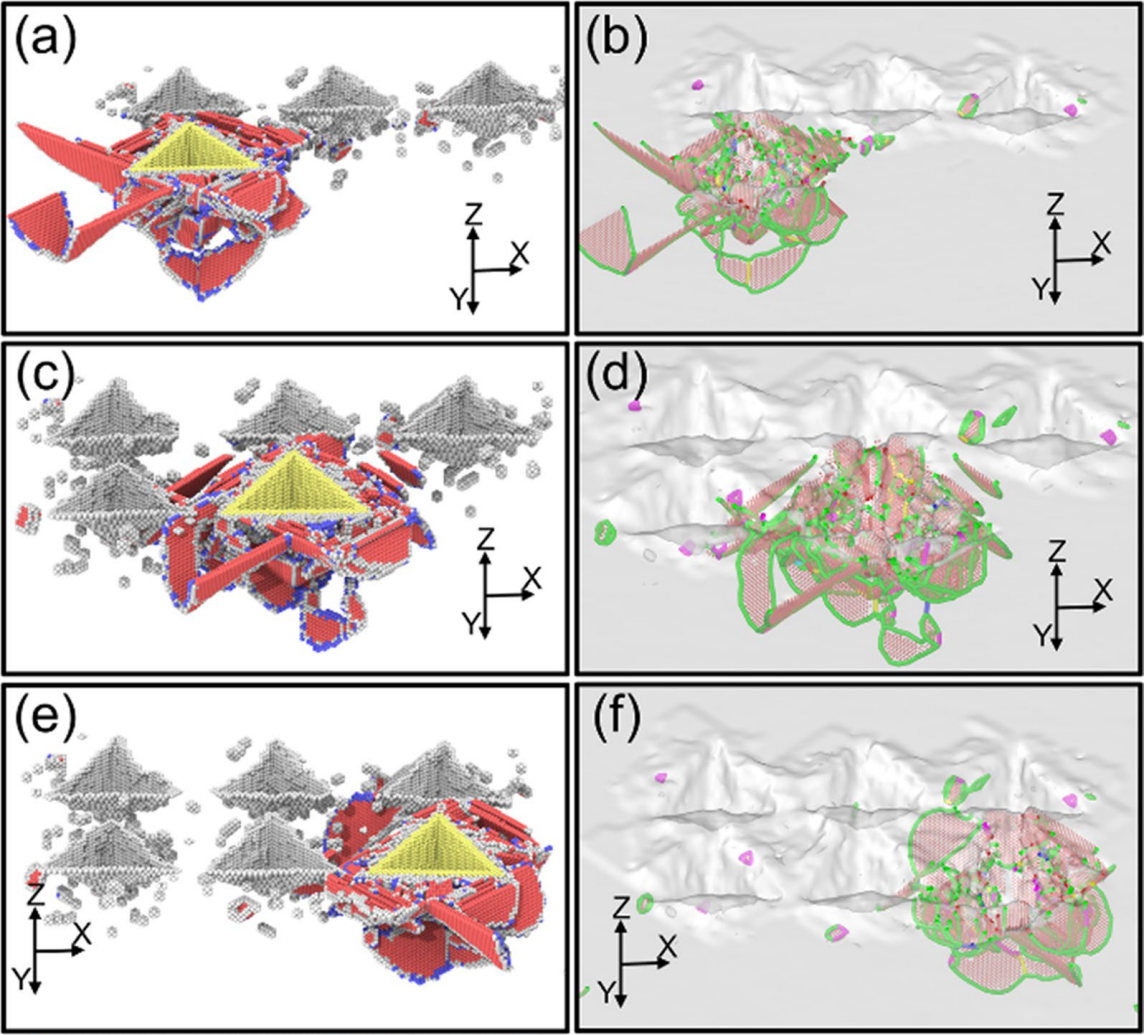
Dislocation structures generated in Single-Indenter indentation process with the spacing of 10 nm **a** CNA snapshot in the fourth indentation, **b** DXA snapshot in fourth indentation, **c** CNA snapshot in the fifth indentation, **d** DXA snapshot in the fifth indentation, **e** CNA snapshot in the sixth indentation, **f** DXA snapshot in the sixth indentation

In Fig. 7, the dislocation distribution exists the difference at the spacing of 10 nm compared with the spacing of 5 nm. As shown in Fig. 7a, b, the degree of the downward dislocation propagation is smaller than the spacing of 5 nm and V-shape dislocations composed of two Shockley-partial dislocations are generated on the left side of the plane OBC. Moreover, the dislocations generated by the surfaces OAC and OAB is less affected by the machined nano-pits. It could be concluded that the various spacing has a great influence on the propagation behavior of the dislocations. When the spacing is larger, the dislocation propagation is less impeded by the processed nano-pits so that the stacking faults are more likely to intersect beneath the indenter which are mainly distributed on the subsurface of the workpiece. When the spacing is small that each probe indentation track overlaps, the movement of the dislocations generated by the extrusion of the surfaces OAC and OAB is hindered by the processed nano-pits. The dislocation loop can be observed beneath the surface OBC.

**Fig. 8:**
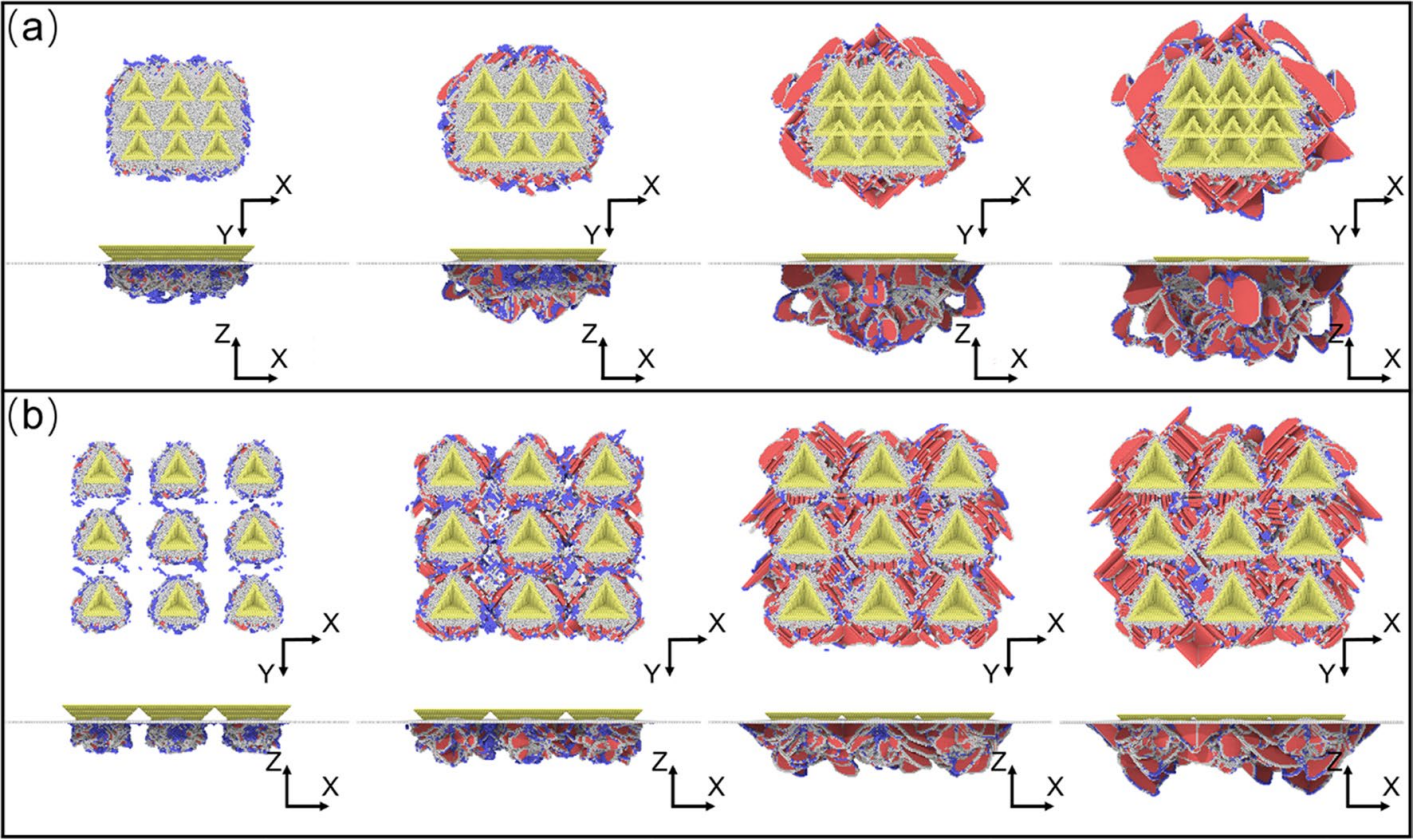
Dislocation structures generated in Indenters-array indentation process **a** CNA snapshots with the spacing of 5 nm, **b** CNA snapshots with the spacing of 10 nm

The defects evolution in the indentation process with an indenter array is shown in Fig. 8. Compared with the indentation process with a single-indenter, the dislocation propagation is well-ordered. Especially in the spacing of 5 nm, the order of defects distribution is significantly improved in Fig. 8a. Due to the small spacing of individual indenters, the dislocations mainly slipped downwards under the loading of the indenter array. When the indenters are completely pressed into the workpiece, the intersecting of each stacking fault prevented from further slipping downward, while the stacking fault distributed around the indenter expands outward. In contrast, the propagation behavior of each indenter is independent in the initial stage of the spacing of 10 nm in Fig. 8b. The stacking faults stop propagating at the earlier stage. Therefore, the extent of the dislocations to slip downward is lower than in the spacing of 5 nm.

## Conclusion

In this study, the indentation processes using a single triangular pyramid indenter and a triangular pyramid indenter array on a single crystal copper specimen are investigated by the MD simulation approach. The machining mechanism for indentation using these two indenters based on the analyses of the indentation morphologies, indentation force and defect evolution during the nano-indentation process is revealed. The main findings can be drawn as follows.

1. The indentation morphologies obtained by single indenter are mainly depended on the spacing of indenters. The shape and consistency of the nano-pit array that machined by the overlapping of indentions are relatively poor when the spacing of indenter is selected as 5 nm. The overlapping effect between indentions can be eliminated when the spacing is 10 nm.
2. A nano-pit array with a better shape and consistency can be machined using the indenter array. The indention force is more stable for using an indenter array compared with using a single indenter. The stacking faults induced by the indenter array are intersected and slipped downward to the specimen when the spacing is chosen as 5 nm. However, when the spacing is selected as 10 nm, dislocations are mainly slipped around the indenter array.
3. The machining efficiency for machining using an indenter array can be improved obviously because several indentions can be obtained simultaneously. Moreover, the alignment problem can be eliminated completely. Thus, the indenter array technique has the potential to be applied in the large-scale production.

Our findings in this study have shown that machining using an indenter array contributes to a nano-pit array with a better shape and consistency and improves the machining efficiency. However, the feature dimension of the MD modelling is too small compared with the practical nanoindentation. A new multi-scale simulation method for nano-metric machining has been proposed recently [27]. Thus, a multi-scale simulation for the nanoindentation process should be conducted and compared with the MD simulation in future work.

## Data Availability

All data generated or analyzed during this study are included in this published article.

## Abbreviations

MD: Molecular dynamic
LAMMPS: Large-scale atomic/molecular massively parallel simulator
HCP: Hexagonal close packed
FCC: Face-center cubic
